# Effects of Selective Enzymatic Hydrolysis on Structural Properties and Gel Properties of Soybean Protein Isolate

**DOI:** 10.3390/foods14223892

**Published:** 2025-11-14

**Authors:** Zhijun Fan, Yue San, Saike Tang, Anhui Ren, Yuejiao Xing, Li Zheng, Zhongjiang Wang

**Affiliations:** 1 College of Food Science, Northeast Agricultural University, Harbin 150030, China; 15845177666@139.com (Z.F.); yuesan2023@163.com (Y.S.); 17532563957@163.com (S.T.); 15205599964@163.com (A.R.); 17743869027@163.com (Y.X.); 2 Heilongjiang Beidahuang Green Health Food Co., Ltd., Kiamusze 154007, China

**Keywords:** enzymatic hydrolysis modification, soybean protein isolate, alkaline protease, papain, structural characteristics, gel properties

## Abstract

Soybean protein isolate (SPI) gel has been demonstrated to exhibit suboptimal stability and a coarse texture. Selective enzymatic hydrolysis modification has been demonstrated to effectively enhance the functional properties and structural stability of the protein. The objective of this study was to modify SPI using alkaline protease and papain. The impact of selective enzymatic hydrolysis on SPI was examined through the analysis of hydrolysis degree (DH), particle size, and protein purity. A systematic exploration was conducted in order to investigate the structural and quality characteristics of SPI gel. Indicators such as secondary structure changes, texture characteristics, water-holding capacity (WHC), rheology, and microstructure were analyzed. The findings indicate that when the DH of the SPI solution is 1%, its particle size is reduced relative to that when DH is 0.5%. The SDS-PAGE results indicated that alkaline protease could hydrolyze most of the 7S and 11S components in SPI into shorter peptides, while papain retained more of the 7S and 11S components and generated peptides with larger molecular weights. Fourier-transform infrared (FT-IR) spectral analysis indicated that following the process of enzymatic modification, the contents of α-helix and β-sheet in the secondary structure of SPI increased, while the contents of β-turns and random coils decreased. In the context of gel performance, it has been demonstrated that papain-modified SPI, attributable to its elevated content of macromolecular peptides, manifests superior WHC, hardness, springiness, cohesiveness, chewiness, storage modulus (G), and microstructure in comparison to alkaline protease-modified gel. Concurrently, the gel performance of papain modified SPI is significantly superior to that of unmodified SPI gel. This research provides a significant theoretical foundation and practical reference for promoting the efficient application of SPI in the domain of food processing.

## 1. Introduction

Proteins play an extremely important role in the development of quality, texture and flavor in many complex food systems, particularly in food systems where protein serves as the primary structural element, such as food gels [1]. Soybean protein isolate (SPI) is a natural plant protein that is rich in multiple essential amino acids, easily digestible, and bioavailable [2]. Due to its high protein content and functional properties, it is widely used in food processing. Additionally, the low cost of SPI can help reduce production expenses to a certain extent. With rising living standards and a growing health consciousness that has shifted dietary habits, SPI has gained global popularity for its diverse applications in vegetarian and low-calorie foods.

Cold-induced Chiba tofu is a protein gel product primarily made from SPI with the addition of transglutaminase (TG), starch, and other auxiliary ingredients [3]. Chiba tofu is renowned for its excellent sensory qualities and strong ability to absorb flavors during cooking. This variety is favored by consumers and is widely popular in Japan, coastal regions of China, and northern China. Furthermore, the stable texture of Chiba tofu enables it to withstand extended cold chain transportation and demanding cooking conditions, thereby significantly expanding its application scenarios. Chiba tofu represents a novel vegetarian food product derived from traditional tofu, offering several advantages over its counterparts. It boasts a high protein content, low carbohydrates, and enhanced texture when cooked—becoming crisper, more elastic, and better at absorbing sauces [4]. However, the product exhibits deficiencies in terms of quality and consistency. Conventionally, the production of tofu entails a process of heat-induced gel, wherein the temperature during gel formation is elevated to a level that exceeds the denaturation point of proteins. This limitation restricts the versatility of the resulting gel. The preparation of Chiba tofu through the use of cold-induced gelation has been demonstrated to be an effective method of overcoming these limitations [5].

Enzymatic modification is a mature and highly efficient protein modification technique that has become an effective means of enhancing the functionality of plant proteins [6]. However, current enzymatic hydrolysis methods lack high selectivity and may fail to accurately release specific peptides that significantly influence gel properties, whereas selective enzymatic hydrolysis offers greater selectivity [7]. Selective enzymatic hydrolysis can change the natural structure of proteins, thereby changing their functional properties, and has the advantages of mild reaction conditions, high specificity and maximum retention of active compounds [8]. Day et al. [9] also demonstrated that selective enzymatic hydrolysis is an effective method for enhancing the functional properties of food proteins while preserving their nutritional value. At present, commonly used enzymes include papain, alkaline protease, bromelain, trypsin, neutral protease, etc. [10]. Compared to other enzymes, papain and alkaline protease offer the advantage of effectively hydrolyzing plant proteins to produce industrially relevant peptides, while being relatively inexpensive [11]. However, extant research has primarily focused on using flavor enzymes to hydrolyze soybean protein for improving its structural properties [12]. The application of alkaline protease treatment to rice proteins has been demonstrated to enhance the functional properties of rice protein protofibrils, thereby improving their stability [13]. In comparison with conventional enzymes, papain hydrolysis of hemp seed protein has been shown to induce the most extensive hydrolysis, yielding minimal peptides with augmented structural flexibility and elevated solubility [6]. However, no studies have explored the modification of SPI using papain and alkaline protease for gel preparation. The present study aims to address the research gap by systematically investigating the effects of enzyme modification on multiple parameters including gel structure, rheological properties, texture, WHC, and microstructure.

Thus, the present study employed a modified SPI, augmented with alkaline protease and papain, as the primary ingredient in the fabrication of cold-induced Chiba tofu. The results of this study demonstrate that gels prepared from modified SPI exhibited superior performance in water retention, gel hardness, springiness, cohesiveness, chewiness, storage modulus (G’) value, and microstructure when compared to unmodified SPI gels. This study provides a foundational technical framework for the development of high-quality cold-induced Chiba tofu. Specifically, the study effectively enhances the quality of the core product through the application of protease-modified SPI. The text continues by providing a foundation of theoretical concepts and practical references for the application of plant proteins in the field of food gelation, emphasizing their potential for high-value uses.

## 2. Materials and Methods

### 2.1. Materials

Soybean protein isolate (SPI) was purchased from Shandong Yuwang Ecological Food Co., Ltd. (Dezhou, Shandong, China). The SPI has a purity of 91.1% and is packaged in 500 g bags. Alcalase alkaline protease was purchased from Solarbio Reagent Company (Beijing, China), with an enzyme activity potency of ≥200 U/mg and a specification of 100 g per bottle. Papain was purchased from Yuan Ye Biotechnology Co., Ltd. (Shanghai, China), featuring an enzyme activity potency of ≥800 U/mg and also available in a specification of 100 g per bottle. Other reagents were purchased from TaiRui Laboratory Supplies Co., Ltd. (Harbin, Heilongjiang, China), with minimum analytical grade.

### 2.2. Determination of Hydrolysis Conditions and Hydrolysis Curves for Alkaline Protease and Papain

We accurately weighed 7.50 g of SPI sample into a conical flask, added 100 mL of distilled water, and prepared a 7.5% (*w*/*w*) SPI solution. The sample was stirred at room temperature using a magnetic stirrer until it was fully dissolved. Subsequently, the sample was immersed in a water bath maintained at 50 °C, and a predetermined amount of alkaline protease/papain enzyme solution was added. The hydrolysis times for alkaline protease were 2, 5, 10, 30, 60, and 120 min, while for papain they were 15, 30, 60, 120, 180, and 240 min. Samples of partial protein solution were collected and transferred into conical flasks. These samples were then subjected to boiling water bath treatment for 10 min to ensure complete enzyme inactivation. After cooling, the sample solution was stored at 4 °C for no more than 1 h. Subsequently, the degree of hydrolysis (DH) of the protein dispersion was determined. Multiple trials were conducted, and a curve was plotted with hydrolysis time on the *x*-axis and DH on the *y*-axis. Through extensive preliminary experiments, we determined that modified with SPI using these two proteases yields optimal results at DH of approximately 0.5% and 1.0%. The hydrolysis conditions corresponding to DH = 0.5% and DH = 1% were identified.

### 2.3. Preparation of Enzyme-Modified Soybean Protein Isolate Samples

Based on the determined hydrolysis conditions, we prepared a large quantity of SPI modified by selective enzymatic hydrolysis. We accurately weighed 30.00 g of SPI sample into a conical flask. We added 400 mL of distilled water and stirred using a magnetic stirrer at room temperature until the sample was completely dissolved. We placed the mixture in a water bath maintained at 50 °C and added an appropriate amount of enzyme solution (alkaline protease and papain). We determined the hydrolysis time based on the respective curves plotted. Immediately after hydrolysis completion, we placed the sample solution in a boiling water bath for thorough inactivation for 10 min. After cooling, we store the sample solution at −18 °C for 12 h. The samples undergo a freeze-drying process using a vacuum freeze-dryer (FD5-3, SIM Corp., Los Angeles, CA, USA).

### 2.4. Determination of Protein Hydrolysis Degree

In the experiment, the degree of protein hydrolysis was determined using the testing method referenced from Liu et al. [14]. The specific procedure was as follows: 10 mL of hydrolyzed protein solution was taken into a beaker, and then 30 mL of CO_2_-free distilled water was added. The sample was mixed thoroughly at room temperature using a magnetic stirrer. 0.1 mol/L NaOH solution was added dropwise to the sample, while the sample’s pH was measured simultaneously using a precision pH meter. When the pH reached 8.2, 10 mL of pre-neutralized formaldehyde solution (pH = 8.2) was added. The volume V_1_ of 0.1 mol/L NaOH solution required to titrate the sample solution to pH = 9.2 was recorded. Distilled water was used as the blank sample, and the volume of NaOH solution used in the blank experiment was denoted as V_2_. The -NH_2_ content of proteins in the sample protein solution was calculated using the following formula:

$$\frac{1000*\mathrm{mol}/L*\left(V_{1}\;-{\;V}_{2}\right)}{10}\left(\mathrm{mmol}/L\right)$$

Hydrolysis degree [15]:

$$\mathrm{DH}=\frac{h}{h_{\mathrm{tot}}}*100\%=\frac{C_{2}\;-\;C_{1}}{7.8\;(\mathrm{mmol}/g)}$$

Among them, C_2_ was the number of NH_2_ in the sample solution after enzymolysis (mmol/L), C_1_ was the number of NH_2_ in the sample solution before enzymolysis (mmol/L). The h_tot_ value represents the total number of peptide bonds in soybean protein, with a value of 7.8 [16].

### 2.5. Determination of Particle Size Distribution

Following the method described by Zheng et al. [17], the protease-modified SPI was dissolved in deionized water to prepare a 1 mg/mL solution, which was stirred at room temperature. The solution was then left to stand overnight at 4 °C to ensure complete dissolution of the sample. The particle size was measured using dynamic light scattering (DLS, Malvern, Panalytical Ltd., Shanghai, China) with a He-Ne laser (633 nm wavelength) at 90°scattering angle. The gel particle size was determined at 25 °C. For each measurement, the average particle size was calculated from 14 measurements and expressed as the volume average diameter D_43_.

### 2.6. Determination of SDS-PAGE 

The sample was dissolved in deionized water to prepare a 2 mg/mL SPI solution. 40 μL of sample was mixed with 2× SDS-PAGE loading buffer (Biyuntian Biotechnology Co., Ltd., Shanghai, China) at a 1:1 (*v*/*v*) ratio. The samples were mixed in a boiling water bath for 5 min to dissolve proteins, then cooled to room temperature (20 ± 2 °C). 20 μL of sample was pipetted for loading. The protein molecular weight standards used are suitable for a molecular weight range of 11 kDa to 245 kDa. During electrophoresis, the concentration gel stage was run at 80 V. When the sample entered the separation gel, the voltage was increased to 120 V and electrophoresis was stopped when the tracer dye reached the bottom of the gel in the horizontal electrophoresis system. After electrophoresis, the gel was stained with Coomassie Brilliant Blue R-250, then decolorized with decolorizing solution (glacial acetic acid: methanol: water = 3:1:36 (*v*/*v*/*v*) [18]. Finally, the protein bands in the gel were scanned using an imager (Gel Doc EZ imager, Bio-Rad, Hercules, CA, USA), and perform quantitative analysis using ImageJ Fiji software [19]. First, use an image conversion tool to adjust brightness and contrast. Second, lanes and bands within each lane were automatically selected with the Lane and Bands tool. Third, use ImageLab’s Lane profile tool to determine the profile of each lane. The volume under the automatically determined peak (corresponding to the detected band’s peak) is manually calibrated by drawing a straight line along the baseline of the profile. Finally, use the analysis table tool to obtain the volume under each peak, which is proportional to the protein quantity.

### 2.7. Preparation of Cold-Induced Gel Samples

Cold-induced gels were prepared using the method described by Zheng et al. [17], comprising four steps. Step one was slurry preparation: 15 g of SPI, 5 g of potato starch, and 80 mL of distilled water were combined in a Braun mixer and stirred at 1000 rpm for approximately 3 min. Transglutaminase (TG) (20 U/g) was then slowly added while the stirring speed was increased to 3000 r/min. Stirring was continued for approximately 2 min until the mixture was thoroughly blended. Next, 0.2 g of NaCl, 0.1 g of monosodium glutamate, and 1 mL of soybean oil were added sequentially at a slow rate. The mixture was then beaten at 9000 rpm for 5 min until the slurry achieved a uniform texture free of fine bubbles. Throughout the entire process, the temperature must be maintained below 12 °C using an ice water bath. The subsequent phase entailed the aging process, which is described in the following section. The process of aging necessitates the meticulous pouring of the slurry into trays, ensuring a thickness of 4 cm is maintained. The surface of the material should be covered with plastic wrap. Ensuring the optimal stability of the protein-starch gel network necessitates the uniform distribution of moisture throughout the gel. This step is crucial for developing a palatable texture. In order to achieve the desired outcome, it is imperative to allow the gel to rest at a temperature of 4 °C for a duration of 10 h. The third step was solidification, in which the matured samples were transferred to a constant-temperature water bath and processed at 50 °C for 45 min. Step four was steaming: When heated, protein molecules cross-link to form a gel network. The temperature around 80 °C is precisely the range where soybean protein begins to coagulate and form a dense, elastic gel structure. This allows tofu to maintain softness while developing a stable “Chiba tofu” texture. Therefore, the coagulated samples are placed in a water bath along with their trays and steamed at 80–85 °C for 40 min, ensuring the product’s core temperature reaches above 75 °C. Finally, after cooling to room temperature, the sample was cut into pieces for later use.

### 2.8. Determination of FT-IR 

The determination of secondary structure was based on the method described by Li et al. [20], with minor modifications. A total of 3 mg of SPI was taken and mixed with KBr powder at a ratio of 1:100 (*w*/*w*), then compressed into a thin film using a tablet press. The sample was scanned 16 times using an FT-IR spectrometer (Nicolet iS10, Thermo, Waltham, MA, USA) across the full wavelength range (4000–400 cm^−1^). The amide I band was observed from 1600 to 1700 cm^−1^. Initially, baseline points were selected at both extremities of the Amide I region for the purpose of linear baseline correction and normalization. Subsequently, Peak Fit v4.12 software was employed to perform deconvolution on the Amide I region, followed by single-peak fitting using a Gaussian function. The proportion of each fitted peak’s area relative to the total Amide I area was calculated, yielding the relative abundance of the corresponding secondary structure. The α-helices are located at 1646–1664 cm^−1^, β-sheets at 1615–1637 cm^−1^ and 1680–1700 cm^−1^, β-turns at 1664–1680 cm^−1^, and random coils at 1637–1645 cm^−1^ [21].

### 2.9. Determination of Textural Properties

Following the test method described by Zhao et al. [22], the hardness, springiness, cohesiveness, and chewiness of the samples were measured at 50% deformation (trigger force of 3 g) using a TA-XT2 Texture Analyzer (Stable Micro Systems Ltd., Godalming, UK) equipped with a P/36R probe (diameter 3.6 cm). The sample is cylindrical in shape with a diameter of 3.6 cm and a height of 2.0 cm. During testing, the initial speed was set to 1.5 mm/s, the test speed to 2.0 mm/s, and the post-compression speed to 2.0 mm/s. Each sample was tested three times. The numbers represent the mean ± standard error (*n* = 3). Hardness values in Pa represent peak stress.

### 2.10. Determination of Water Holding Capacity

Determined the WHC of the sample according to the method described by Sun et al. [23]. Took a gel sample (10 g) into a 50 mL centrifuge tube, then centrifuged at 4 °C and 3000× *g* for 15 min. After removing water, weighed the sample and calculated the gel’s WHC using the following formula:

WHC (%) = (W_1_ − W_2_)/W × 100

where W is the weight of the sample, W_1_ is the total weight of the sample and centrifuge tube before centrifugation, W_2_ is the total weight of the sample and centrifuge tube after water removal.

### 2.11. Determination of Dynamic Rheological Properties

The rheology of the samples modified by selective enzymatic hydrolysis was tested according to the method of Chang et al. [24], with minor modifications. The samples were analyzed using a DHR-3 rheometer (TA Instruments, Waters Technology Corporation, Milford, MA, USA) equipped with parallel plates (diameter 40 mm, gap 1 mm) in temperature-scanning mode. The linear viscoelastic region was verified through strain scanning. Test specimens were uniformly prepared as 2 cm diameter, 3 mm thick sheets. The digested sample was thoroughly mixed with the enzyme using a vortex shaker, then injected between the parallel plates. When the plates were filled with the solution, the excess sample was removed. The surface of the sample was coated with silicone oil and covered with a protective cap. During testing, the maximum strain was set to 0.01, with a constant oscillation frequency of 1 Hz. Starting at 25 °C, the sample was heated at 2 °C/min to 95 °C and held at 95 °C for 20 min. It was then cooled at 2 °C/min back to 25 °C while recording parameters including the storage modulus (G’) and loss modulus (G”).

### 2.12. Scanning Electron Microscope Analysis

The morphology and microstructure of the gel were determined according to the method described by Lin et al. [25]. First, we cut the gel into small pieces (2 × 5 mm), fixed them for 4 h in 0.1 M sodium phosphate buffer (pH 6.8) containing 2.5 M glutaraldehyde, and then stored them overnight in a refrigerator (4 °C). We rinsed twice with the above buffer solution for 15 min each time. Dehydration was achieved using a series of graded ethanol solutions (50%, 70%, and 90%), with each rinse lasting 15 min. The sample was dehydrated twice with 100% ethanol, each rinse lasting 15 min. The sample was then rinsed for 15 min in a 1:1 volume mixture of 100% ethanol and tert-butanol, followed by a 15 min rinse in pure tert-butanol solution. Subsequently, the gel samples were subjected to freeze-drying. Subsequent to the application of a gold coating with a thickness of 8 nm to the sample surface, observations of the microstructure were conducted utilizing a scanning SEM (XL-30 ESEM FEG, FEI Company, Hillsboro, OR, USA). Images were captured at an acceleration voltage of 5 kV and a magnification of 3000×.

### 2.13. Data Analysis

Each experiment was repeated three times, and all results were expressed as mean ± standard deviation. Graphs were created using Origin 2018 software, and statistical analysis was performed using one-way analysis of variance (ANOVA) and Duncan’s multiple range test, completed with SPSS 19.0 software (*p* < 0.05, Significant difference).

## 3. Results and Analysis

### 3.1. Effects of Selective Enzymatic Hydrolysis Modification on Structural Characteristics of Soybean Protein Isolate

#### 3.1.1. Hydrolysis Curves of Alcalase Alkaline Protease and Papain Hydrolysis

**Figure 1:**
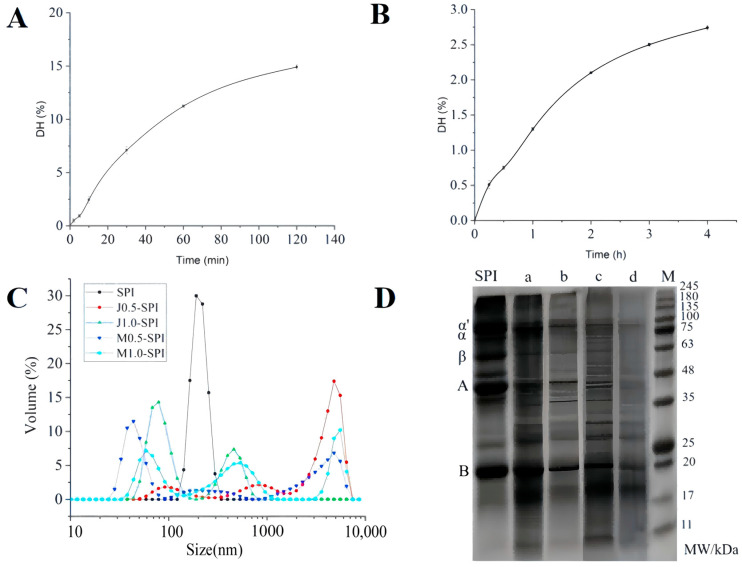
The hydrolysis curves of soybean protein isolate treated with alkaline protease and *Papain* (**A**,**B**), respectively. The error bars in the figure represent the standard deviation of each data group (n = 3). The effect of enzymatic modification on the particle size distribution of soybean protein isolate (**C**). SPI denotes untreated gel, J0.5-SPI, J1.0-SPI, M0.5-SPI and M1.0-SPI denote gels hydrolyzed SPI by Alcalase and papain with hydrolysis at 0.5% and 1.0%, respectively. SDS-PAGE profile of enzymatically modified SPI (**D**). M denotes protein marker, SPI denotes SPI control group, (a,b) denote DH = 0.5% and DH = 1% papain modified SPI, (c,d) denote SPI modified with alkaline protease at DH = 0.5% and DH = 1%.

The DH was determined with Alcalase alkaline protease added at a level of 5 mg per gram of substrate. The curve shown in Figure 1A was plotted with hydrolysis time on the *x*-axis and DH on the *y*-axis. After extensive preliminary trials, the following results were obtained: SPI hydrolyzed for 2 min yielded a DH of 0.49%, SPI hydrolyzed for 5 min yielded a DH of 0.93%, SPI hydrolyzed for 10 min yielded a DH of 2.44%, SPI hydrolyzed for 30 min yielded a DH of 7.09%, SPI hydrolyzed for 60 min yielded a DH of 11.23%, SPI hydrolyzed for 120 min yielded a DH of 14.9%. As shown in the figure, the reaction rate of Alcalase hydrolysis gradually levels off as the hydrolysis time increases. Compared to papain, alkaline protease exhibits the broadest range of hydrolysis, achieving the highest DH within a short time frame. This leads to significant unfolding of soybean protein structure, exposure of hydrophobic residues, and increased free sulfhydryl groups [26]. During the enzymatic hydrolysis process, Alcalase first specifically cleaves peptide bonds within the SPI molecule, preferentially targeting bonds formed by hydrophobic amino acid residues [27]. This process induces conformational unfolding of the SPI molecule, exposing potential reaction sites such as sulfhydryl groups while simultaneously triggering the rearrangement of the protein’s secondary and tertiary structures. As the reaction progresses, the resulting peptide fragments compete with the active site, leading to a decrease in the enzymatic reaction rate. The hydrolysis process proceeds at a slow rate, enabling DH to be controlled by adjusting the hydrolysis time, thereby achieving selective hydrolysis of the SPI molecules. The final hydrolysis conditions for Alcalase alkaline protease-modified SPI are as follows: substrate concentration 7.5%, original pH, temperature 50 °C, enzyme loading 400 U/g.

The DH was determined, with papain added at a level of 5 mg per gram of substrate. A curve was plotted with hydrolysis time on the *x*-axis and DH on the *y*-axis, as shown in Figure 1B. Papain, a cysteine endonuclease with broad specificity, at target sites such as lysine (Lys), arginine (Arg) and phenylalanine (Phe), is particularly effective in increasing DH of proteins [28]. After extensive preliminary trials, the following results were obtained: SPI hydrolyzed for 15 min yielded a DH of 0.51%, SPI hydrolyzed for 30 min yielded a DH of 0.75%, SPI hydrolyzed for 60 min yielded a DH of 1.3%, SPI hydrolyzed for 120 min yielded a DH of 2.1%, SPI hydrolyzed for 180 min yielded a DH of 2.5%, and SPI hydrolyzed for 240 min yielded a DH of 2.74%. Yin et al. [29] found that hydrolyzing soybean protein with trypsin and papain, respectively, resulted in an increase in DH as hydrolysis time increased, with papain consistently yielding higher DH values than trypsin. This is consistent with the experimental results, that is, the hydrolysis of SPI is positively correlated with the hydrolysis time. As shown in the figure, the reaction rate of papain hydrolysis gradually levels off as the hydrolysis time increases. The hydrolysis process proceeds at a slow rate, enabling control of DH through hydrolysis time, thereby achieving selective hydrolysis of SPI molecules. The final hydrolysis conditions for papain-modified SPI are as follows: substrate concentration 7.5%, original pH, temperature 50 °C, enzyme loading 25 U/g.

#### 3.1.2. Preparation of Enzyme-Modified Soybean Protein Isolate Products

Using linear interpolation, DH values were first measured at various time points. With hydrolysis time as the *x*-axis and DH as the *y*-axis, a linear interpolation equation was obtained to determine the required hydrolysis times for enzyme-modified SPI at DH = 0.5% and DH = 1%. When DH = 0.5% and 1.0%, mild hydrolysis cleaves only a small number of peptide bonds, causing localized unfolding of protein chains and exposing more hydrophilic groups. These groups form numerous hydrogen bonds with water molecules, thereby enhancing water absorption and retention capacity while also improving gel hardness, springness, and cohesiveness. When DH > 1.0%, the product’s emulsifying and foaming properties are enhanced [30]. From the alkaline protease hydrolysis curve of SPI in Figure 1A, calculations indicate that the hydrolysis times required for preparing Alcalase-modified SPI with DH = 0.5% and DH = 1% are 2.2 min and 4.5 min, respectively. From the papain hydrolysis curve of SPI in Figure 1B, calculations indicate that the hydrolysis times required for preparing papain-modified SPI with DH = 0.5% and DH = 1% are 19 min and 45 min, respectively. Based on the determined enzyme modification conditions, prepare enzyme-modified SPI samples. After hydrolysis completion, inactivate the samples by boiling water bath treatment for 10 min. Allow the sample solution to cool, then store it at −18 °C for 12 h. Freeze-dry the frozen samples using a vacuum freeze-dryer. Sublimation drying was conducted for 48 h at 25–35 °C under a vacuum of 1 Pa to ensure complete removal of water. The final DH of the enzyme-modified samples was determined, with results showing that the DH values for Alcalase-modified SPI are 0.51% and 0.98%, respectively, while those for papain-modified SPI are 0.52% and 1.02%, respectively. Therefore, it can be approximated that their hydrolyzation degrees are approximately 0.5% and 1%, respectively.

#### 3.1.3. Determination of Particle Size and Distribution

The effects of different types of selective enzymatic hydrolysis modification on the particle size and distribution of SPI solutions were analyzed and tested using dynamic light scattering technology. As shown in Table 1, selective enzymatic hydrolysis modification significantly affects the particle size distribution of SPI solutions. Research indicates that alkaline protease modification reduced the particle size of SPI solution from 2311.33 nm to 424.93 nm, while papain modification decreased it from 2311.33 nm to 241.1 nm. The particle size of the sample with a hydrolysis rate of 1% is smaller than that of the sample with a DH of 0.5%, and the particle size of the papain modified sample is smaller than that of the basic protease modified sample at the same DH, and this difference may be caused by the different modes of action of different types of enzymes [31]. Papain hydrolysis (which is primarily surface-limited) rapidly breaks down protein molecules into short-chain oligopeptides, preventing the original macromolecules from intertwining and aggregating. In contrast, deep endotomy by alkaline proteases generates numerous hydrophobic fragments within the protein, which tend to self-assemble in aqueous solutions, forming larger particles [32].

Figure 1C shows the particle size distribution of sample solutions for Alcalase and papain-selectively hydrolyzed modified SPI at DH = 0.5% and DH = 1%. From the figure, it can be seen that the untreated SPI is distributed in a single peak, and the samples modified by enzymatic digestion show a multi-peak state, the transition from a unimodal to a multimodal particle size distribution is a fingerprint of the enzymatic dismantling of SPI’s aggregated structure. It reveals a process where large, polydisperse aggregates are selectively cleaved into a mixture of distinct populations: resistant cores, soluble oligomers/large peptides, and small peptides. The specific shape and evolution of these peaks are governed by the enzyme’s specificity and DH, ultimately dictating the new functional properties of the modified protein ingredient. The sample particle size shifts to the left in the direction of small size, with the increase in hydrolysis, the distribution of particles becomes more concentrated, which may be due to the fact that after protein hydrolysis, the peptide bonds are broken, some subunits are dissociated, and the small molecule particles increase and the particle size becomes smaller [33].

#### 3.1.4. SDS-PAGE Analysis

Figure 1D shows the SDS-PAGE profile of SPI modified by selective enzymatic hydrolysis. As shown in the figure, both papain and alkaline protease-modified SPI samples produce numerous low-molecular-weight peptide fragments with molecular weights below 20 kDa. Following selective hydrolysis with Alcalase, the α’, α, and β subunits of SPI all exhibit a tendency toward substantial hydrolysis. Compared to samples with DH = 0.5%, the degradation of polypeptide subunits is more extensive at DH = 1%. In samples of both types of DH, the acidic subunit of 11S exhibits a hydrolyzed state, with hydrolysis becoming more complete as DH increases. For papain, which acts on the α’, α, and β subunits, some regions remain unhydrolyzed, particularly in modified samples with DH = 0.5%. The acidic subunit of 11S protein also exhibits a phenomenon where most of it remains undegraded, and when papain DH = 0.5%, even less degradation occurs. Compared to samples modified with Alcalase, samples modified with papain exhibit a greater distribution of proteins and peptide fragments above a molecular weight of 35 kDa. By analyzing and comparing the SDS-PAGE profiles of SPI modified by papain and Alcalase alkaline proteases, it can be further inferred that differences in enzyme types result in varying catalytic specificities. Alcalase is a non-specific peptidase, whereas papain exhibits greater specificity. At low DH, the final enzymatic modification patterns of SPI differ accordingly [34]. Alcalase possesses multiple catalytic sites, leading to the hydrolysis of most 7S and 11S components in the modified sample and yielding shorter peptide fragments. The catalytic sites of papain exhibit a preference for hydrophobic and aromatic residues [35]. The modified samples retained relatively higher proportions of the 7S and 11S fractions, yielding larger peptide fragments [36]. It can be predicted that differences in enzymatic hydrolysis patterns resulting from variations in enzyme types will exert different effects on the functional properties of modified SPI samples.

#### 3.1.5. FT-IR Analysis

FT-IR is an effective method for analyzing protein secondary structure [37]. Eight peaks were obtained by analyzing the amide I spectrum using second-derivative curve convolution. Based on the assignment of corresponding peaks, the proportions of various secondary structures in the sample were calculated as shown in Table 2. As shown in the table, the primary structural feature of both the SPI sample and the selective enzymatic hydrolysis modification SPI sample is the β-sheet structure. Following alkaline protease hydrolysis, the proportions of α-helix and β-sheet increase from 16% and 50.1% to 18.84% and 55.76%, respectively. The β-turn and random coil content decrease from 14.1% and 19.8% to 13.12% and 12.27%, respectively. Papain hydrolysis exhibits the same trend, particularly when the DH reaches 0.5%, with the β-sheet content increasing to 57.55% compared to untreated SPI [38]. When the DH reaches 1.0%, the β-sheet content decreases to 56.85%. In our study, the increase in α-helix content after enzymatic hydrolysis may be caused by the transition from disordered to ordered structure of random coil [39]. Following the addition of TG for cross-linking in the gel, the increase in β-sheet structure content may be attributed to the hydrophobic groups within the papain-exposed protein molecules being cross-linked by TG into a dense gel network structure [40]. This finding is consistent with the previously measured changes in gel strength. Therefore, it can be concluded that modification with alkaline protease and papain alters the secondary structure of proteins and promotes TG cross-linking into a dense gel network structure.

### 3.2. Effect of Selective Enzymatic Hydrolysis Modification on the Gel Properties of Soybean Protein Isolate

#### 3.2.1. Texture Characteristic Analysis

The texture of the protein gel is crucial for evaluating the quality of Chiba tofu gel [41]. Analysis results indicate that both the enzyme-modified SPI gel and the control SPI gel exhibit significant changes in properties. Li et al. [26]. experimental results indicate that selecting an enzyme concentration of 0.5–1.0%, temperature of 55 °C, pH of 6.5–7.0, and incubation for 2–4 h achieves a dual effect of “partial hydrolysis and cross-linking,” significantly influencing the final gel’s hardness, springiness, water retention, and sensory texture. The control SPI cold-induced gel demonstrates low springiness values, low gel strength and forms an opaque gel state. The gel texture was significantly improved after modification by different enzymes. The textural properties of SPI gels modified by alkaline protease and papain are shown in Table 3. As can be seen from the table, when papain hydrolyzes SPI with DH = 0.5%, the gel exhibits maximum hardness (damage resistance) of 6.32 Pa, but when the DH of papain increases, the hardness of the gel decreases. Alkaline protease and papain exhibit the same trend. The springiness, cohesiveness, and chewiness of the gel all decrease as the DH of different enzymes increase. The increased DH may be attributed to the disruption of the primary structure of protein molecules, resulting in shorter molecular chains that hinder gel formation. Consequently, the gel strength of enzymatically modified SPI decreases [33]. The gel strength of papain-modified SPI, whether at DH = 0.5% or DH = 1%, is significantly higher than that of alcalase-modified SPI. This clearly demonstrates that different enzymatic modification patterns lead to distinct functional properties. Papain exhibits high affinity for hydrophobic and aromatic residues, cleaving only at select sites, thus preserving most peptide bonds. The modified SPI contains a higher proportion of macromolecular fragments, forming a network dominated by hydrophobic interactions. During hydrolysis, internal hydrophobic groups are exposed, providing numerous accessible covalent bond sites for TG cross-linking. This forms a dense three-dimensional network structure, significantly enhancing gel strength [42]. Conversely, Alcalase, with its numerous cleavage sites, produces modified SPI containing relatively more small-molecule fragments and consequently lower gel strength [43]. Papain-modified SPI gel exhibits enhanced strength, resulting in firmer and more springiness Chiba tofu. The intact secondary structure of macromolecular fragments enables the formation of uniform, fine pores within the tofu, delivering a smoother texture that meets consumer expectations.

#### 3.2.2. Gel Water-Holding Capacity Analysis

**Figure 2:**
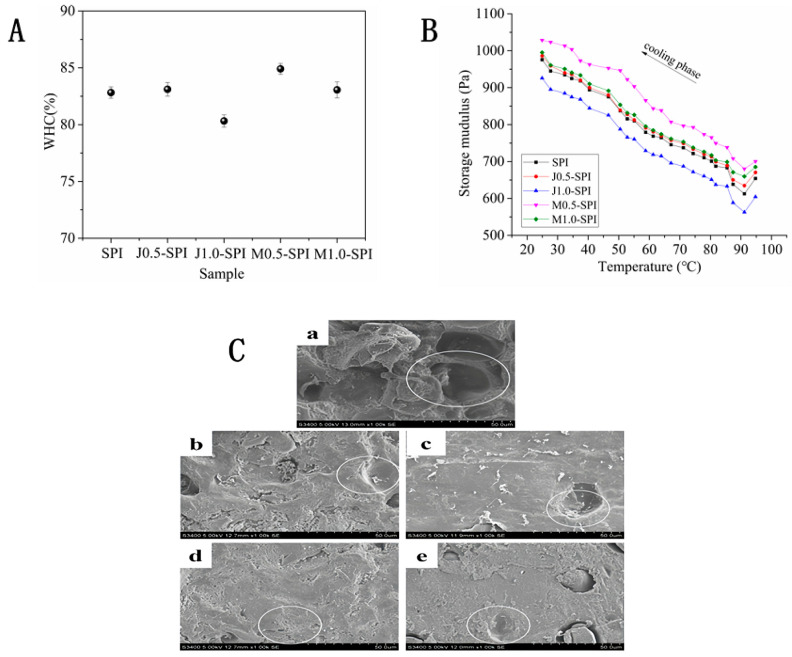
The effect of enzyme modification on the WHC of soybean protein isolate gel (**A**). SPI denotes untreated gel, J0.5-SPI, J1.0-SPI, M0.5-SPI and M1.0-SPI denote gels hydrolyzed SPI by alkaline protease and papain with hydrolysis at 0.5% and 1.0%, respectively. Storage modulus (G’) of TG cross-linked Chiba tofu gel after partial hydrolysis of papain and alcalase during the cooling phase (**B**). The effect of enzyme modification on the microstructure of soybean protein isolate gel (**C**). (**a**) denotes untreated gel, (**b**,**c**) denote SPI modified with alkaline protease at DH = 0.5% and DH = 1%, (**d**,**e**) denote DH = 0.5% and DH = 1% papain modified SPI.

WHC of gels demonstrates the interaction between proteins and water, and also serves as an important criterion for evaluating gel quality [44]. As shown in Figure 2A, when papain hydrolyzed SPI with DH = 0.5%, the WHC value of the gel reached its maximum of 85%. Zheng et al. [34] investigated the gel properties of composite gels formed from soybean protein isolate and silver carp protein after papain treatment. The study found that the most significant effect occurred when the SPI with DH = 0.5%, at which point the WHC reached 80%, demonstrating remarkable water retention performance. Both studies confirm the positive effect of enzyme modification on gel WHC under low hydrolysis conditions. However, the 85% WHC achieved in this research exceeds the values reported by Zheng et al. As early as 2004, Pallarès et al. [45] demonstrated that enzymatic hydrolysis can relax protein structures while exposing hydrophobic groups within the protein molecules. The specific cleavage of lysine residues by papain can expose additional lysine sites. When TG enzyme is added during the subsequent formation of cold-induced gels, it crosslinks into a dense, structured gel network. This tightly packed and uniform microstructure binds water within the gel system, thereby enhancing gel strength and WHC performance. Gels with higher WHC can more effectively capture moisture when forming a three-dimensional network, thereby enhancing the gel’s springness and overall texture while strengthening its cohesiveness [46]. Furthermore, Kao et al. [47] also indicates that a dense and uniform microstructure of protein gels corresponds to higher gel strength and WHC. The WHC of the hydrolyzed gel in this study exhibited the same trend as the gel strength mentioned above, further supporting this conclusion. In our study, when DH reaches 1%, the gel hardness and WHC begin to decrease. This decline may be attributed to excessive hydrolysis of proteins into smaller peptides, which is detrimental to TG cross-linking.

#### 3.2.3. Rheological Analysis

The gelation behavior of TG-crosslinked cold-induced gels modified by alkaline protease and papain selective enzymatic hydrolysis modification was investigated using a rheometer (temperature scanning). The gel formation and gelation capabilities were evaluated through dynamic rheology. The storage modulus (G’) reflects the solid properties of the system and is related to the springiness of the gel [48]. The dynamic rheological data for samples modified with alkaline protease and papain are shown in Figure 2B. As the formed protein network structure gradually becomes stable and irreversible, the G’ values of all samples increase with time during the cooling scan phase [49]. The degree of gel hydrolysis is positively correlated with enzyme concentration. It exhibits an exponential relationship with processing time, with the highest hydrolysis rate occurring during the initial 2–4 h. Beyond 4–6 h, the hydrolysis rate increases only slowly. As the DH by alkaline protease and papain increases, the G’ value gradually decreases. When papain DH = 0.5%, the G’ value peaks at 1028.6 Pa. When the alkaline protease and papain DH are 0.5%, the degree of protein cleavage increases with rising enzyme dosage. Intramolecular hydrophobic groups are progressively exposed, forming a more compact network structure. However, as the protease dosage continues to increase, the side chains of proteins undergo excessive hydrolysis. As the net charge increases, the enhanced electrostatic repulsion between peptides reduces the protein’s gelation ability [50], leading to a decrease in the G’ value. Moreover, Sun & Arntfield [51] indicated that the protein gel network structure is primarily maintained and strengthened through hydrophobic interactions and hydrogen bonds during the cooling scanning phase, suggesting that the cross-linking reaction induced by TG predominantly occurs during the cooling stage. When the temperature finally drops to 25 °C, alkaline protease selectively hydrolyzed SPI. G’ values of the gels prepared at 0.5% and 1% hydrolysis levels are 986.1 Pa and 925.8 Pa, respectively. G’ values of the gels prepared with papain-selected hydrolyzed SPI at 0.5% and 1% are 1028.6 Pa and 995.3 Pa, respectively, while the value of the SPI gel in the control group is 975.8 Pa. Therefore, when papain is used to hydrolyze SPI at a DH of 0.5%, the gel exhibits the highest G’ value (1028.6 Pa), consistent with the results of textural property measurements. The higher the G’ value, the tighter the protein gel network and the greater the hardness. Simultaneously, tofu with a high G’ value exhibits superior springiness, allowing it to rebound quickly after compression. When the G’ value is excessively high, the tofu surface becomes coarse and the texture slightly hard.

#### 3.2.4. Microstructural Analysis

Figure 2C shows the microstructure of TG-crosslinked cold-induced gels after enzymatic hydrolysis with Alcalase alkaline protease and papain at concentrations of 0.5% and 1%. As shown in the figure, the pores in the unmodified SPI gel control group (Figure 2C(a)) are significantly larger than those in the protease-modified SPI gel samples (Figure 2C(b–e)). Notably, the gel sample hydrolyzed by papain with DH = 0.5% exhibits a compact network structure (Figure 2C(d)). The reason for these phenomena may be that unenzymatically hydrolyzed proteins retain their original compact folding, making it difficult for internal hydrophobic groups to be exposed. This results in fewer intermolecular interaction points, leading to the formation of networks with large pore sizes and irregular shapes [52]. However, as the DH increases, the gel’s network structure becomes larger. The cause of these phenomena is that protease hydrolysis breaks the disulfide bonds in SPI, stretching its structure to expose more hydrophobic groups and free sulfhydryl groups [31]. This promotes TG cross-linking into a dense gel network. However, as the degree of enzymatic hydrolysis increases, SPI may undergo excessive hydrolysis into smaller peptides, which is detrimental to the formation of a dense TG-crosslinked gel network structure. The dense gel network structure can bind more water. Our SEM analysis of the gel is consistent with the findings of Puppo & Añón [53]. The latter indicates that protein gels with dense, uniform microstructures exhibit higher WHC than those with coarse, loose microstructures. This trend is consistent with our previous findings on gel texture, WHC, and rheological parameters.

## 4. Conclusions

This study employed selective enzymatic hydrolysis to modify SPI, systematically investigating the effects of protease types and DH on SPI structure and gel properties. The results indicate that the particle size of the solution at a DH of 1% was smaller than that at a DH of 0.5%. SDS-PAGE analysis revealed that following alkaline protease modification, the majority of the 7S and 11S components in SPI are hydrolyzed, yielding shorter peptide fragments. In contrast, papain-modified SPI retains relatively more 7S and 11S components, resulting in larger peptide fragments. This is because alcalase, which prefers to hydrolyze peptide bonds adjacent to hydrophobic amino acid residues, exhibits the characteristic of “rapid and deep hydrolysis”, papain only targets peptide bonds adjacent to basic amino acid residues and features “mild and partial hydrolysis”. This allows it to retain more core structures of the 7S and 11S components and generate large-sized peptides, thereby laying the foundation for the differentiation of gel functions. Enzymatic hydrolysis enhances the order of SPI through hierarchical structural regulation. Specifically, it starts with the number of peptide bond cleavages in the primary structure (quantified by DH value), followed by an increase in the proportion of ordered structures such as α-helices and β-sheets in the secondary structure (FT-IR analysis results), and finally leads to differences in particle size in the aggregated structure. This process forms a “structural modification cascade effect” and provides support for the construction of gel networks. The higher content of large peptide fragments in papain-modified SPI contributes to its superior gel performance, it exhibits superior performance compared to Alkaline protease-modified gel and unmodified SPI gel in terms of WHC, gel hardness, springiness, cohesiveness, chewiness, G′ value and microstructure. When papain hydrolyzed SPI at a DH of 0.5%, the gel achieved a WHC of 85%, a maximum hardness of 6.32 Pa, and a peak G’ value of 1028.6 Pa. These findings provide a strategic blueprint: papain-hydrolyzed SPI is ideal for structured foods like plant-based meat analogs, while extensively hydrolyzed SPI suits liquid nutrition. Therefore, selective enzymatic hydrolysis enables precise engineering of SPI functionality for diverse food applications.

## Figures and Tables

**Table 1:** The effect of selective enzymatic hydrolysis on the particle size of soybean protein isolate.

Sample	D_43_ (nm)	PDI
SPI	2311.33 ± 40.00 ^a^	0.81 ± 0.04 ^a^
J0.5-SPI	444.50 ± 60.00 ^b^	0.78 ± 0.02 ^ab^
J1.0-SPI	424.93 ± 30.00 ^b^	0.75 ± 0.01 ^bc^
M0.5-SPI	263.43 ± 40.00 ^c^	0.73 ± 0.02 ^c^
M1.0-SPI	241.10 ± 10.00 ^c^	0.72 ± 0.03 ^c^

Note: SPI denotes untreated gel, J0.5-SPI, J1.0-SPI, M0.5-SPI and M1.0-SPI denote gels hydrolyzed SPI by alkaline protease and papain with hydrolysis at 0.5% and 1.0%, respectively. The numbers represent the mean ± standard error (n = 3), different lowercase letters within the same column indicate significant differences, *p* < 0.05.

**Table 2:** The contents of secondary structures of gel products.

Sample	α-Helix (%)	β-Sheet (%)	β-Turn (%)	Random Coil (%)
SPI	16.00 ± 0.10 ^d^	50.10 ± 1.10 ^c^	14.10 ± 0.10 ^a^	19.80 ± 1.00 ^a^
J0.5-SPI	18.15 ± 0.32 ^b^	55.70 ± 0.40 ^b^	13.32 ± 0.32 ^b^	12.83 ± 0.13 ^bc^
J1.0-SPI	18.84 ± 0.23 ^a^	55.76 ± 0.76 ^ab^	13.12 ± 0.52 ^b^	12.27 ± 0.20 ^c^
M0.5-SPI	16.67 ± 0.43 ^c^	57.55 ± 0.55 ^a^	12.81 ± 0.26 ^b^	12.97 ± 0.62 ^bc^
M1.0-SPI	16.09 ± 0.10 ^d^	56.85 ± 0.33 ^ab^	13.40 ± 0.10 ^b^	13.66 ± 0.51 ^b^

Note: SPI denotes untreated gel; J0.5-SPI, J1.0-SPI, M0.5-SPI and M1.0-SPI denote gels hydrolyzed SPI by alkaline protease and papain with hydrolysis at 0.5% and 1.0%, respectively. The numbers represent the mean ± standard error (n = 3), different lowercase letters within the same column indicate significant differences, *p* < 0.05.

**Table 3:** The effect of selective enzymatic hydrolysis on the texture properties of soybean protein isolate gel.

Sample	Hardness/Pa	Springiness	Cohesiveness	Chewiness/Pa
SPI	2.76 ± 0.07 ^c^	0.75 ± 0.04 ^c^	0.91 ± 0.01 ^ab^	1.88 ± 0.14 ^c^
J0.5-SPI	5.40 ± 0.20 ^b^	0.82 ± 0.01 ^a^	0.92 ± 0.02 ^a^	4.07 ± 0.20 ^b^
J1.0-SPI	2.68 ± 0.03 ^c^	0.76 ± 0.01 ^b^	0.87 ± 0.04 ^b^	1.77 ± 0.30 ^c^
M0.5-SPI	6.32 ± 0.02 ^a^	0.86 ± 0.02 ^a^	0.94 ± 0.03 ^a^	5.11 ± 0.10 ^a^
M1.0-SPI	5.36 ± 0.02 ^b^	0.84 ± 0.03 ^a^	0.93 ± 0.02 ^a^	4.19 ± 0.20 ^b^

Note: SPI denotes untreated gel, J0.5-SPI, J1.0-SPI, M0.5-SPI and M1.0-SPI denote gels hydrolyzed SPI by alkaline protease and papain with hydrolysis at 0.5% and 1.0%, respectively. Different lowercase letters within the same column indicate significant differences, *p* < 0.05.

## Data Availability

The original contributions presented in this study are included in the article. Further inquiries can be directed to the corresponding authors.
